# Imaging Dynamic Molecular Signaling by the Cdc42 GTPase within the Developing CNS

**DOI:** 10.1371/journal.pone.0088870

**Published:** 2014-02-19

**Authors:** Nima Sharifai, Hasitha Samarajeewa, Daichi Kamiyama, Tzyy-Chyn Deng, Maria Boulina, Akira Chiba

**Affiliations:** 1 Department of Biology, University of Miami, Coral Gables, Florida, United States of America; 2 Miami Institute of Molecular Imaging and Computation, Coral Gables, Florida, United States of America; 3 Department of Pharmaceutical Chemistry, University of California, San Francisco, California, United States of America; National Institutes of Health (NIH), United States of America

## Abstract

Protein interactions underlie the complexity of neuronal function. Potential interactions between specific proteins in the brain are predicted from assays based on genetic interaction and/or biochemistry. Genetic interaction reveals endogenous, but not necessarily direct, interactions between the proteins. Biochemistry-based assays, on the other hand, demonstrate direct interactions between proteins, but often outside their native environment or without a subcellular context. We aimed to achieve the best of both approaches by visualizing protein interaction directly within the brain of a live animal. Here, we show a proof-of-principle experiment in which the Cdc42 GTPase associates with its alleged partner WASp within neurons during the time and space that coincide with the newly developing CNS.

## Introduction

Protein interactions are physical events that take place at nanometer scales. Dissociation constants indicate that non-covalent bonds that form between the interacting proteins can have a million-fold affinity advantage over casual encounters. However, within each cell of an intact organism, the probability of protein association and hence signaling changes continually through diverse posttranslational modifications. For example, a substantial portion of interactome is impacted by binary activations in a large array of monomeric GTPases [1] as well as by extensive phosphorylation and dephosphorylation of divers proteins [2]. Due to a shortage of methods capable of uncovering its dynamics, the time and place of individual molecular signaling remains undetermined *in vivo* for a majority of cases. In neuroscience, decades of research progress have been hampered by the difficulties of bridging molecular explanations to cellular neurobiological phenomena [3]. The question of when and where a particular pair of interacting proteins engages in physical association is rarely investigated on the same experimental platform as the question of how it might contribute specifically to synaptogenesis or any other aspects of neuronal differentiation. To address this challenge, we sought to visualize protein interactions directly in their native environment by utilizing Förster resonance energy transfer (FRET) [4]. Such an approach, i.e., interpreting the intermolecular FRET as proxy for protein-protein interaction, could circumvent the need to characterize both known and unknown plasticity of protein behavior in response to changes in immediate cellular environment. In this study, by combining transgenics [5], [6] with an imaging technology [7], [8], we quantitate protein interactions by Cdc42 (cell division control protein 42 homolog) and its alleged signaling partner WASp (Wiskott–Aldrich Syndrome protein) within the native environment of a developing brain.

## Results

### Signaling Proteins within Neurons

**Figure 1:**
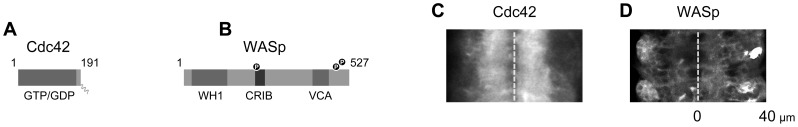
Cdc42 and WASp. A. Cdc42 becomes active when it replaces GDP with GTP. B. WASp receives phosphorylation and can associate with active Cdc42 through its CRIB domain. C Localization of Cdc42 (*elav-GAL4/UAS-mCherry::Cdc42*). D Localization of WASp2 (*elav-GAL4/UAS-mCherry::WASp*). *Dashed line* indicates midline in the CNS segment.

The GTPase Cdc42 (Fig. 1A) is thought to contribute to complex morphogenesis of neurons [9]. Genetic deletion of Cdc42 in *Drosophila* results in both presynaptic and postsynaptic defects manifested toward the end of neurogenesis in the embryo [10], [11]. However, the protein’s continual presence in the neuronal cytosol pauses a challenge as to how this ubiquitously-expressed versatile signal protein has a function that is highly restricted in time and space *in vivo*. Part of this is thought to reflect Cdc42’s endogenous activation being concentrated during the later stages of neurogenesis [11]. This evolutionarily conserved signal protein is kept inactive within neurons prior to their axonal extension and dendritic formation, i.e., until about 9 hours before the completion of embryogenesis. Cdc42’s activation pattern within the nervous system is, nevertheless, considerably more widespread compared to where its knockout phenotype emerges. This raises the possibility that factors other than the Cdc42’s activation might further limit its signaling within the neurons. Biochemistry-based assays have isolated an array of cytoplasmic proteins as Cdc42’s potential binding partners [12], [13]. Among these, WASp (Fig. 1B) is implicated in cytoskeletal and membrane dynamics within the axons and/or dendrites of neurons [14]. Essential to animal survival [10], [15], both Cdc42 and WASp are expressed ubiquitously throughout neurogenesis [16]. WASp receives extensive phosphorylation that potentially modulates its ability to interact with activated Cdc42. However, whether WASp binds Cdc42 *in vivo*, let alone when and where Cdc42 would signal through this specific partner within the neurons remain unknown.

### FRET as Proxy for Protein Interaction

**Figure 2:**
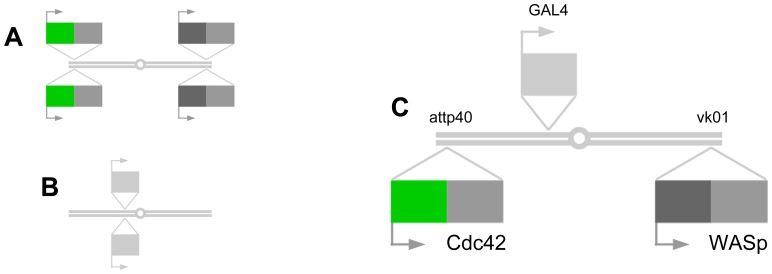
Low-dosage expression. A. Transgene stock carrying two GAL4-responsive transgenes (for example *UAS-mEGFP::Cdc42* and *UAS-mCherry:WASp*). B. GAL driver stock (*elav-GAL4*). C. Experimental animal with single transgene dosages.

**Figure 3:**
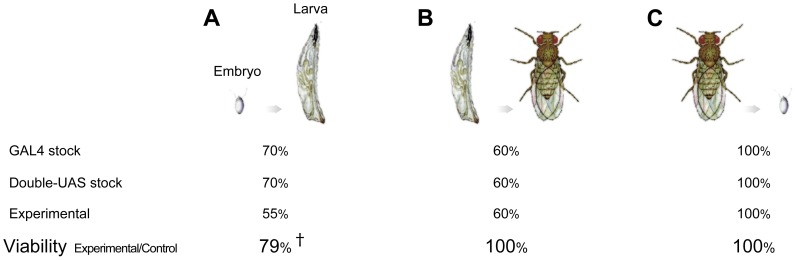
Minimum artefacts. A. Embryo to larva survival rate. Suspected as an early-stage lethality (†). B. Larva to adult survival rate. C. Adult fertility and expected offspring genotype occurrence rate. Viability is the survival rate of the experimental animals as compared to both parental controls.

When fluorescence donor and acceptor molecules come within a distance of approximately 9 nm from each other, the donor’s fluorescence lifetime decreases as a result of FRET [7]. We chose monomeric mEGFP [17] and mCherry [18] as the donor and acceptor, respectively. Their separate phylogenic origins make it unlikely to form a dimer by themselves. With their transparency, anatomical compactness and genetic manipulability, *Drosophila* embryos offer unparalleled opportunities to visualize various cellular and molecular events within a whole organism without requiring dissection or fixation [19]. In order to express Cdc42 and WASp as fluorescently-labeled proteins at a reproducible low dosage, we designed expression vectors (Deng et al., unpublished) carrying both GAL4-responsive *UAS* and phiC31-dependent *attp* recognition sequences [5], [6]. Under a single cell type-specific driver, the GAL4/UAS system allows for expressing two fluorescently labeled proteins within the same cells. The site-specific integration of the transgenes with phiC31 integrase further eliminates any position-dependent variability that might arise in the transgene expressivity. Crossing the stocks thus produces embryos expressing both *UAS-mEGFP::Cdc42* and *UAS-mCherry::WASp* each at a single transgene dosage in all of neurons under *elav-GAL4* driver (Fig. 2). Previous study with genetic replacement confirmed that mEGFP tagging of Cdc42 can be considered functionally benign [11]. Thus, we were able to monitor the interaction of the protein pair *in vivo* with not only minimal but also precisely controlled artefacts expected from expressing them as fluorescently labeled exogenous proteins (Fig. 3).

There exists a fundamental difference between the intermolecular FRET, which we seek to detect, and the intra-molecular FRET that is designed into many biosensors [20], [21]. The latter cases have both the donor and acceptor of FRET encoded within a single polypeptide chain. The FRET pair’s maximum distance, then, is two foreign protein domains away from each other – still a FRET-able distance. Users only need to differentiate a high-level FRET from a low-level FRET that occurs in the same molecules. In contrast, we aim to detect FRET that might or might not occur between two fluorescently-labeled proteins that are separately introduced into the cytosol. The weight of negative and positive controls in such experiments is substantial.

**Figure 4:**
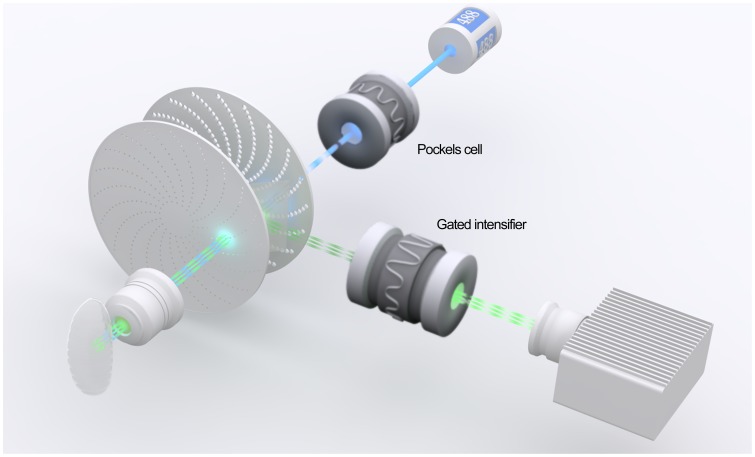
Direct molecular imaging in a live animal. FRET detection within a live whole *Drosophila* embryo by 3D frequency-domain FLIM (fluorescence lifetime imaging microscope). *Pockels cell* electro-optic modulator and *gated intensifier* are synchronized at approximately 100 MHz. The relatively low light intensity facilitates efficient averaging of the lifetimes from individual fluorescently-labeled proteins in a large number of confocal pixels.

**Figure 5:**
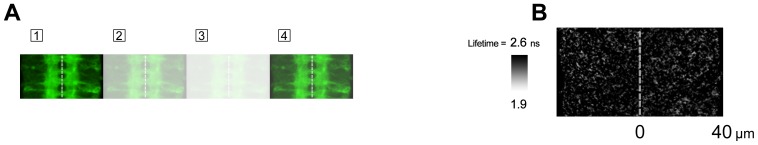
Lifetime quantification. A. mEGFP emission is collected at four phase-shifted points. B. Mean fluorescence lifetime in a CNS segment examined (see *A*).

Measuring the donor fluorescence lifetime is a reliable way to quantify FRET within cells *in vivo* [22]. Therefore, we adopted 3D frequency-domain fluorescence lifetime imaging microscope (FLIM) [8] to measure the fluorescence lifetime of mEGFP (Fig. 4). Utilizing a low-intensity light with a spinning disk cofocal unit on CCD-based fluorescence lifetime imaging components, this microscope allows for an efficient detection of an average donor lifetime in the entire image field (Fig. 5). The fluorescence lifetime is a concentration-independent value, circumventing the issue of variable expression levels within the neurons that can compromise ratiometric FRET calculations [8], [24]. Using this, we validated that FRET can be detected between the fluorescently labeled Cdc42 and WASp, a well-known interaction partners, within the compact nervous system of the intact *Drosophila* embryo.

**Figure 6:**
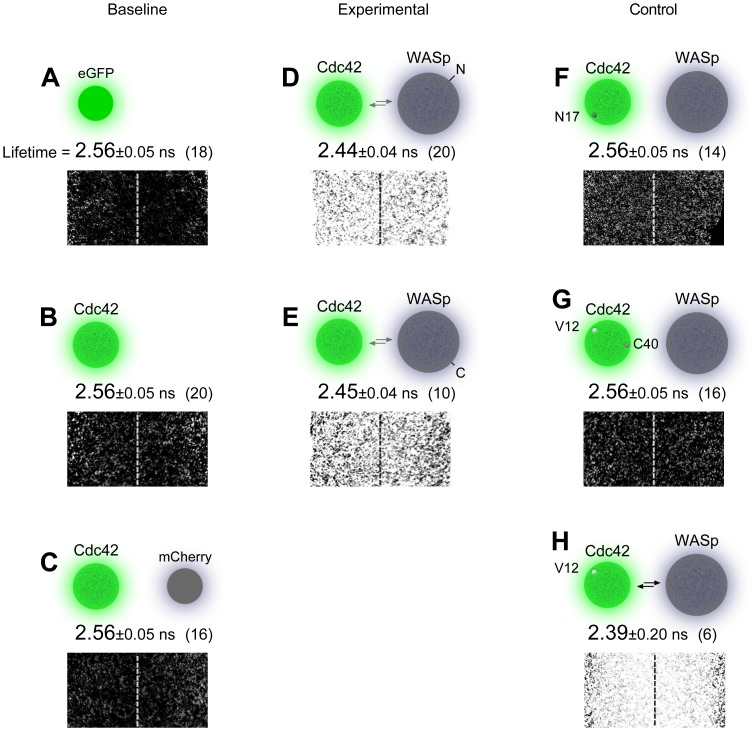
Sensitivity to detect protein-protein interaction. A mEGFP lifetime by itself. B. As used as tag for Cdc42. C. With co-expression of cytoplasmic mCherry. D–E. FRET is detected as a drop in donor fluorescence lifetime with co-expression of WASp tagged with mCherry at amino (*D*) or carboxyl (*E*) terminus. F–G. Point mutations in Cdc42 prevent it from associating with WASp. H. A point mutation in Cdc42 promotes its associating with WASp. Each protein (pair) displays a mean lifetime (mean±s.d.) in *n* embryos at hour 15 (*see* Fig. 5 for scale bar).

When expressed alone in the entire nervous system, mEGFP exhibits a constant fluorescence lifetime of 2.56 ns, independent of its local concentration (Fig. 6A). We measured the mEGFP lifetime after fusing it to Cdc42 with a short flexible linker at its amino-terminus, and obtained the same fluorescence lifetime of 2.56 ns (Fig. 6B). Adding WASp, instead of Cdc42, to mEGFP also resulted in the lifetime of 2.56 ns (not shown). Therefore, when used as a tag to label a specific protein, mEGFP exhibited a constant fluorescence lifetime. Having established this, we added mCherry to the neuronal cytosol as a separate protein. If interaction occurs between two proteins that are either fluorescent or fluorescently-labeled, the donor fluorescence lifetime will drop. In this baseline experiment, however, neither mEGFP nor Cdc42 tagged by mEGFP was anticipated to bind mCherry. The mean mEGFP fluorescence lifetime we obtained was 2.56 ns (Fig. 6C). We define this as ‘baseline’ fluorescence lifetime for a mEGFP-labeled protein.

We next measured the fluorescence lifetime of mEGFP, the FRET donor, when Cdc42 and WASp were labeled with mEGFP and mCherry, respectively. We saw the mean donor fluorescence lifetime drop to 2.45 ns (Fig. 6D). We repeated this experiment by labeling WASp at its carboxyl terminus, instead of its amino terminus. There we obtained a similar donor lifetime of 2.44 ns (Fig. 6E). When we photo-bleached mCherry, mEGFP exhibited the baseline lifetime value (not shown). To address sample-to-sample variability, we repeated with 10–20 embryos in each experiment and the drop from the baseline was significant at p<0.001 with t-test.

Several mutations in Cdc42 are known to prevent this protein from binding to its partners. Since there is, at least formally, a small likelihood that an unknown molecule is mediating the FRET between mEGFP-labeled Cdc42 and mCherry-labeled WASp, we sought to use inactivating mutant forms of Cdc42s to test molecular specificity of interaction. One is a single amino acid substitution G17N, which retains Cdc42 at its GDP-bound inactive state [9]. When we replaced the wild type Cdc42 with this mutant Cdc42, the donor lifetime reverted to 2.56 ns (Fig. 6F). Another is a double point-mutation G12V-Y40C that only impairs Cdc42’s ability to bind a specific subset of its effectors, i.e., those that contain Cdc42/Rac1 interactive binding (CRIB) domains [24]. This second mutant also retained the donor lifetime at 2.56 ns with WASp (Fig. 6G). Having the G12V mutation alone, on the other hand, Cdc42 exhibited the FRET at a level noticeably higher than wild type Cdc42 (Fig. 6H). Based on these, we conclude that the interaction detected through FRET *in vivo* between Cdc42 and WASp is direct.

### Cdc42 and its Partner Interact *in vivo*

**Figure 7:**
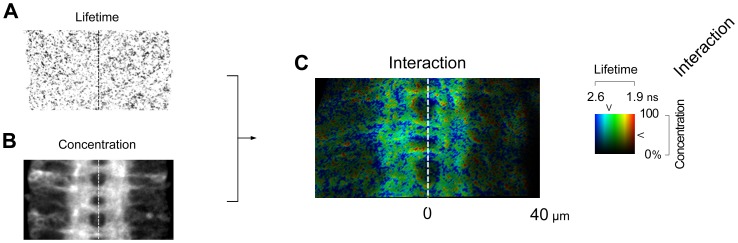
Spatial resolution of interaction. A. Lifetime is concentration-independent. B. Concentration varies from pixel to pixel and requires correction for FRET. C. Interaction is a product of lifetime and concentration.

**Figure 8:**
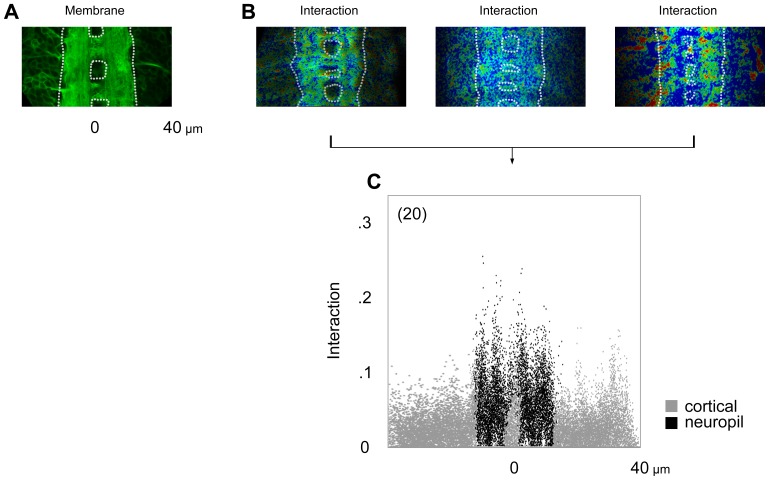
Pixel-resolution analysis. A. Neuropil emerges near the center of the CNS as a plasma membrane-enriched region by hour 21 (*dotted line*). B. Samples from a given experiment show both variability and consistency. C. Pixoplot displays individual pixels from multiple samples (*n*) along the axes of interaction and spatial position within the bilateral CNS. *Black* color indicates pixels with neuropil assignment.

Both Cdc42 and WASp are expressed in the *Drosophila* nervous system from very early on during embryogenesis. However, the interaction between Cdc42 and WASp was found to be spatially and temporally limited. We calculated the extent of protein-protein interaction at individual pixels of each image. This resulted in spatially rich data with about eight thousand pixels in a single segment of the CNS (Fig. 7). As noted, the FLIM collects the mean lifetime value from all fluorophores present in a given pixel, which can be used to calculate the proportion of donor molecules undergoing FRET (see below). Using mEGFP intensity, it also quantifies the amount of the donor molecules present. Software applies necessary correction for the fluorescence loss due to FRET. Interaction, then, is a product of the mean donor fluorescence FRET efficiency lifetime and the corrected quantity of the donor molecules present. We plotted individual pixels along the axes of interaction and position within the CNS (Fig. 8). Pixel colors indicate their assignment as either within or outside the neuropil. This offered a convenient way to summarize data from multiple embryos of a given experiment.

**Figure 9:**
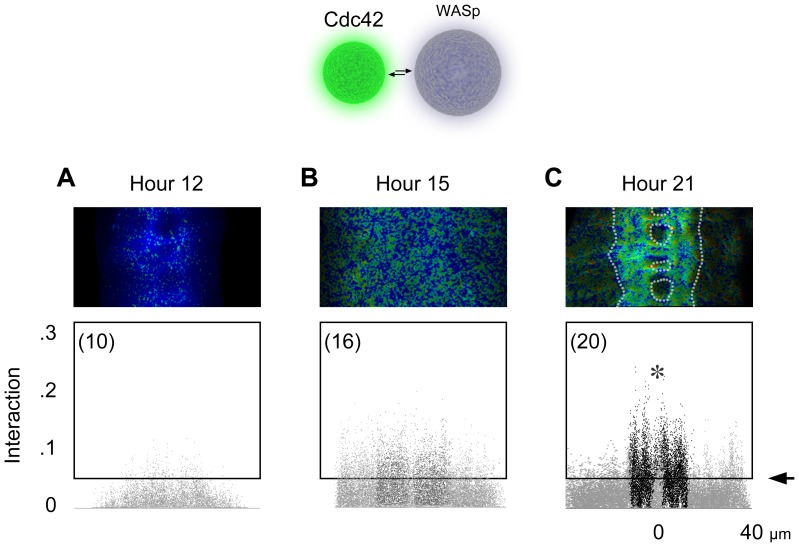
Cdc42-to-WASp interaction. A–C. Interaction between Cdc42 and WASp before (*A*), at the onset of (*B*), and after the formation of neuropil (*C*). *Arrow* points to the threshold based on background fluorescence.

At hour 12, when the CNS was narrow, little interaction occurred (Fig. 9A). By hour 15, the CNS increased its volume and pro-neuropil appeared near the center of the CNS (Fig. 9B). The Cdc42-to-WASp interaction became noticeable at this point but with only a small difference between those pixels within pro-neuropil and those outside. Over the subsequent hours, this pro-neuropil would transform into the neuropil, rich in plasma membrane. At hour 21, as the embryogenesis neared its completion, the spatial pattern of interaction became dramatic (Fig. 9C). Neuropil pixels within the longitudinal connectives, approximately from 5 to 15 µm on both sides of the midline, had the largest degree of interaction (Fig. 9C *asterisk*). Thus, the Cdc42-to-WASp interaction in the neurons became apparent after hour 15, with its peak arriving several hours later in the center of the nerve cord, the neuropil, where axon terminals and dendritic branches intermingle.

**Figure 10:**
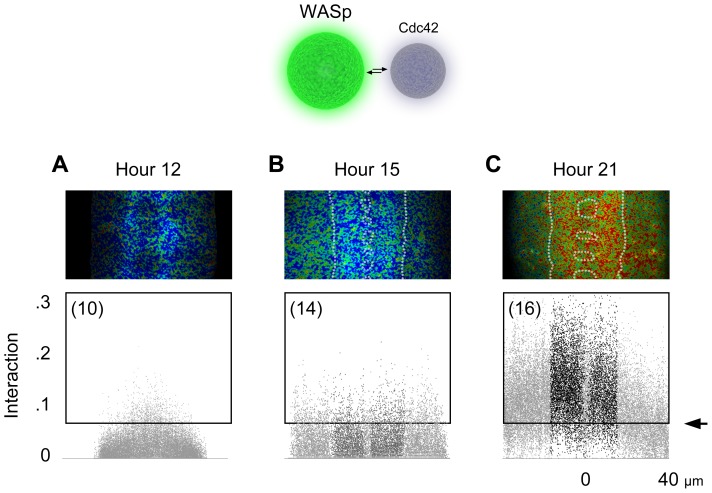
WASp-to-Cdc42 interaction. A–C. Interaction between Cdc42 and WASp before (*A*), at the onset of (*B*), and after the formation of neuropil (*C*).

**Figure 11:**
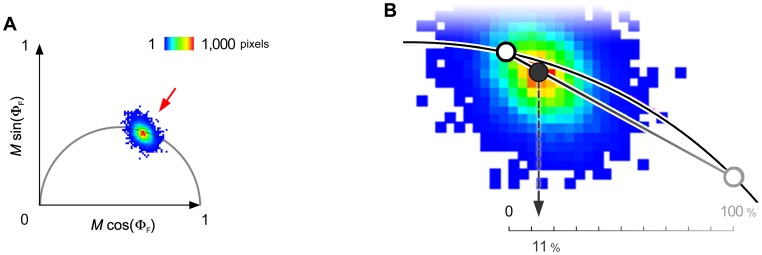
Percentage of interaction. A. All pixels from an image are plotted on a single Polarplot. Averages of fluorescence lifetime in individual pixels cluster around a ‘mean’ lifetime (*red arrow*). B. The known baseline lifetime, i.e., 2.56 ns, defines 0% FRET percentage on the semicircle (*black open* circle). A straight line projecting through the ‘mean’ lifetime (*black* circle) intersects with the semicircle at 100% FRET percentage (*grey open* circle). The position of the ‘mean’ in respect to the 0% and100% FRET is the estimate of percentage of interaction between the pair of proteins examined.

While Cdc42 concentration was high in the neuropil, WASp’s was relatively low (compare Fig. 1C to 1D). We noted, nevertheless, that both proteins were present in all pixels of neurons throughout the entire nervous system. This meant that the interaction between Cdc42 and WASp occurred only in a fraction of places and times in which the two proteins were co-localized. Therefore, we repeated the FRET measurement using the reciprocal tagging, i.e., mEGFP labeled WASp and mCherry, Cdc42 (Fig. 10). The resulting pattern of FRET was very similar overall, although the fraction of WASp interacting with Cdc42 (∼30%) was approximately three times larger than the fraction of Cdc42 interacting with WASp (∼10%). Polarplot [26] allows for a theoretical estimate of the fraction of the fluorescently-labeled Cdc42 binding the fluorescently-labeled WASp (Fig. 11). This observation might reflect that Cdc42, being a signaling hub, has more interaction partners than does WASp and, hence, a smaller portion of the Cdc42 pool binds to WASp than *vice versa*.

We propose that Cdc42 interacts with WASp at the time and place that coincides with the development of the functional CNS at this stage – most likely involved in dendrite elaboration, fine glial wrapping of axons and/or synapse formation within the neuropil.

## Discussion

### From Molecules to Neurons to a Brain

Networks evolve because their links change dynamically over time and space. In this work, we showed changes in a specific link in the protein-protein interaction network within the developing brain through a method that offers spatial and temporal resolution. Of all pixels that exhibited co-localization of Cdc42 and WASp, approximately a half may feature Cdc42 in its active state [11]. However, only a small fraction of those pixels showed Cdc42 interacting with its partner WASp. This fraction, nevertheless, happened to be highly coincidental to the formation of synapse-enriched neuropil in this developing nervous system. Thus, when predicting where a knockout phenotype might emerge within the animal, the knowledge of where the protein interacts with its partner conveys more value than where they are present. Future studies could look at both downstream and upstream signaling partners of Cdc42 and WASp and, using different GAL4 drivers, characterize their pathways within a single developing neuron. Unlike biochemistry-based assays [26], [27], the direct imaging described here does not compromise tissue or cellular integrity. It is a quantitative approach capable of bridging the nanometer-scale molecular circuitry to the micrometer-scale neuronal connectivity within the developing brain.

## Materials and Methods

### A. Transgenic Resource

We constructed transgenic GAL4-responsive lines that label Cdc42 with mEGFP, the donor of FRET, and WASp with mCherry, the acceptor of FRET. mEGFP and mCherry together yield Förster radius of 5.4 nm [28]. With a fast decay by the sixth power of the increasing distance between donor and acceptor fluorophores, this translates to FRET being detectable up to 9.1 nm. The two fluorescent proteins are among the best genetically encoded FRET pairs available. Both can mature rapidly at 25°C, a normal temperature for *Drosophila* experiments. Photo-stable as imaging agents, they are also non-toxic to cells. Derived from evolutionarily discrete protein families of jellyfish and coral, respectively, the pair has no propensity to oligomerize. Because we intend to determine when and where the proteins being labeled by these fluorescent proteins associate physically, any inherent fluorophore affinities could confound our ability to determine this. Both mEGFP and mCherry are red-shifted as compared to, for example, CFP-YFP pair and would allow for a superior tissue penetration depth while producing less background by light. To minimize the experiment-to-experiment variables and also keep artefacts at a low level if any, we combined several transgenic technologies. Frist, the cDNA’s were sequence-validated clones from the Berkeley *Drosophila* Genome Project. Second, we prepared a set of expression vectors designed to label proteins of interest (POI) at their amino terminus with either mEGFP or mCherry. An additional set was made to tag POIs at their C-terminus. In all cases, we used a short flexible linker sequence. Third, we adopted site-directed phiC31 integrase to insert the transgenes to specific loci in the *Drosophila* genome. We targeted all mEGFP-containing transgenes to *attp40* (25C6) site and all mCherry-containing transgenes to *vk01* (59D3) site. This facilitated recombining them into double-transgene stocks. Using these allowed us to express different protein pairs in a GAL4-positive cell population [5]. With the *elav-GAL4* driver, we delivered two single-copy transgenes (for example, *UAS-mEGFP::Cdc42* and *UAS-mCherry::WASp*) in all neurons. Due to the deliberately low expression level, we did not note that any of these labeled proteins compromise the animals’ viability. The viability showed no significant disadvantage from larval through adult stages. Even their fertility rate turned out the same as the control, and transgene fidelity in the offspring was as expected.

### B. Imaging Tool

FLIM used in this study combines micrometer spatial resolution of fluorescence imaging with nanosecond temporal resolution of fluorescence lifetime. Whereas intensity-based FRET imaging measurements require the determination of several parameters and corrections of artefacts, FLIM does not require these and, thus, is a highly reliable way to determine the FRET values. As compared to previously described time-domain FLIM in which high-energy pulsed laser is employed to illuminate each pixel multiple times, photo-damage in our system was virtually non-existent and, furthermore, the speed of data acquisition was faster. These features were advantageous for measuring the lifetime of genetically encoded fluorescent proteins such as mEGFP in biological samples. FRET leads to both energy loss from donor and corresponding gain by acceptor. FLIM quantifies the change in the donor’s fluorescence lifetime. In many FRET-based biosensors in which the donor and acceptor are tethered in a single polypeptide, ratiometric sensitized emission quantification methods are suitable and could even be economical. However, when the local concentration of the donor and/or the acceptor is either unknown or difficult to measure, as in most biological samples, the change in the lifetime of the donor’s fluorescence that does not depend on concentration is considered to be the best, if not the only, method through which the FRET can be quantified reliably [7]. FLIM collects the observed lifetime for all fluorophores present in a given pixel. Being capable of averaging the mean of a large number of lifetimes from individual fluorescently-labeled proteins simplified the data even when each protein-protein interaction could have occurred for a very brief duration. Unlike in a cuvette, however, biological samples have an unknown number of macromolecules that absorb and, sometimes, fluoresce at non-uniform concentrations. High-quality confocal imaging is thus essential, yet the data acquisition time must not be overly long. The proteins that produce FRET could translocate or the tissue might begin to deteriorate. Without dissection, we were able to collect an image of 696×520 pixels, capturing the mean per-pixel donor fluorescence lifetime at a single focal plane of the CNS of a *Drosophila* embryo. This translated to our not having to fix the samples, an effective way to eliminate a major source of artefacts.

#### Acquisition

Images were acquired using a custom-assembled frequency-domain upright FLIM system from Intelligent Imaging Innovations Inc. (3i). A continuous–wave laser modulated using Pockels cell electro-optic modulator, was synchronized with a CoolSnap EZ camera using a Lambert Instruments II18MD intensifier in this method of FLIM. Yokogawa CSU-X1 was used for fast image acquisition with a Zeiss W Plan-Apochromat 63x (n.a. 1.0) water-immersion objective lens. Semrock 440/521/607/700 emission filter was used with Semrock Di10 T488/568 diochroic as the emission pathway. Image intensification was maintained at 2800 units across all experiments. To calibrate the system, a pH-sensitive fluorophore 1-hydroxypyrene-3,6,8-trisulfonate (HPTS) solution was used as standard for 5.4 ns. Images were taken in a focal plane where the embryo’s CNS possessed maximal neuropil width. Exposures were set for the channels to create an intensity dynamic range ∼75% for CCD capture. On average, 2.0–4.0 seconds of total exposure was needed to collect four images with different phase-shifts from a given sample.

#### Interaction

The extent of protein-protein interaction is a product of the mean donor fluorescence lifetime and the quantity of the donor molecules present, with the latter quantity being corrected and normalized to the maximum value within the image. It is defined as: *I* = *FE*⋅*C_corr_,* where *I* is the interaction between a pair of proteins in focus, *FE* represents the FRET efficiency for a given pixel. C_corr_ is a relative estimate of its mEGFP concentration provided by comparing the pixel’s intensity to maximal pixel intensity in the image: *C_cor_*_r_ = *I_G_/I_max_*, where *I_G_* and *I_ma_*_x_ are corrected for intensity lost to FRET by I^*^ = *I*/(1-*FE*). While FRET efficiency alone can indicate the proportion of mEGFP molecules undergoing FRET in a given pixel or pixels, the ‘Interaction’ equation signifies the quantity of mEGFP molecules undergoing FRET in that pixel(s). The result is an ability to assess spatial hotspots, where the most FRET events are occurring, within the CNS and determine whether these locations vary at different developmental time points. Pixoplots were based on a pixel-by-pixel analysis with each pixel carrying a contextual assignment such as within or outside an anatomically defined region. Interaction values calculated were plotted against the absolute value of the perpendicular distance from each pixel to a predefined midline of the CNS as the x axis. Threshold in the plot is determined by the lifetime standard deviation among pixels in a donor-only sample, which allowed us to select a minimum FRET efficiency value that exceeds the value of >95% of pixels. Hence, threshold is defined by the calculated minimum FRET efficiency (0.087 for Cdc42, 0.095 for WASp) multiplied by the mean intensity value (0.475 for Cdc42, 0.701 for WASp).

#### FRET percentage

While FRET efficiency captures the extent of energy transfer between two fluorophores, it will not reflect the percentage of donor molecules undergoing FRET if the transfer does not reach 100% efficiency. Polarplot analysis, however, makes it possible to calculate this latter value, i.e., fraction of mEGFP-labeled proteins interacting with mCherry-labeled proteins. The polarplot histogram gives a sample’s mean lifetime value along with its XY coordinates on the plot [26]. A donor fluorophore with monoexponential decay can have two lifetime states, i.e., baseline over no FRET or FRET. However both will have coordinates that fall somewhere on the semicircle. Because a given sample, region, or pixel features many individual fluorophores, the mean lifetime value and its coordinates will reflect a heterogeneous population. Nevertheless, those coordinates will follow a linear trajectory between those of the baseline and FRET states, and the % of donor molecules undergoing FRET can be calculated as: % donors FRETing = (*τ_s_* × *d_b-s_*)/(( *τ_s_* × *d_b-s_*)+( *τ_s_* × *d_s-f_*)), where *τ_s_* is the mean lifetime of the sample, and *d_b-s_* and *d_s-f_* are the distances between the X Y coordinates of the baseline-to-sample and sample-to-full FRET, respectively, and d = ((*x_1_– x_2_*)^2^+ (*y_1_– y_2_*)^2^)^1/2^.
